# Behavior and Early-Age Performance of Continuously Reinforced Concrete Bus Pad

**DOI:** 10.3390/ma18133143

**Published:** 2025-07-02

**Authors:** Sang Cheol Park, Kang In Lee, Soon Ho Baek, Sang Jin Kim, Seong-Min Kim

**Affiliations:** Department of Civil Engineering, Kyung Hee University, Yongin 17104, Republic of Korea; scpark555@khu.ac.kr (S.C.P.); leerkddls123@khu.ac.kr (K.I.L.); qortnsgh1@khu.ac.kr (S.H.B.); kai7290@khu.ac.kr (S.J.K.)

**Keywords:** bus pad, bus stop pavement, continuously reinforced concrete pavement, environmental load, moving vehicle load, slab, steel bar

## Abstract

The behavior of the cast-in-place continuously reinforced concrete (CRC) bus pad applied to bus stop pavement in a central bus-only lane was experimentally analyzed under environmental and moving vehicle loads, and the early-age performance of the CRC bus pad was evaluated using experimental data and finite element analysis results. Using various measurement sensors, the concrete slab strain, longitudinal steel bar strains, horizontal and vertical displacements, and crack behavior of the CRC bus pad due to environmental loads were measured, and the dynamic responses of the concrete slab and steel bars due to moving vehicle loads were also measured. Additionally, a method for converting strain gauge measurements of a cracked concrete slab to the strain of an uncracked concrete slab was also proposed. Under environmental loads, the range of stresses acting on the steel bars and the bond between concrete and steel bars were analyzed to be appropriate for ensuring excellent performance of the CRC bus pad. The crack widths and vertical and longitudinal displacements of the CRC bus pad were found to have no effect on the pavement performance. Within the vehicle velocity range used in this experiment, the strains of the slab and steel bars as the vehicle passed through the CRC bus pad were virtually independent of the vehicle velocity and were within a range that did not cause any reduction in pavement performance. This study confirmed that the CRC bus pad has excellent performance and can replace asphalt concrete bus stop pavement or jointed concrete bus pad.

## 1. Introduction

**Figure 1:**
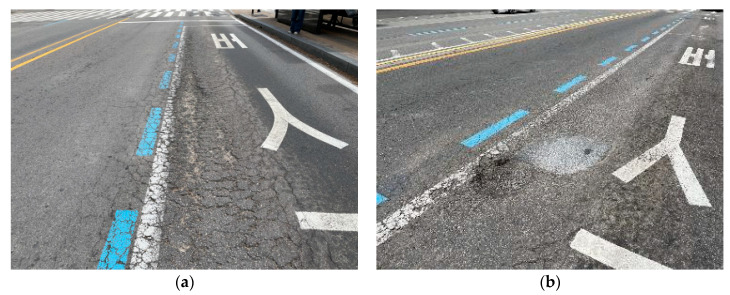
Damages of bus stop asphalt concrete pavement: (**a**) cracks and rutting; (**b**) pothole.

As populations in urban areas increase, the number of users of public transportation is rapidly increasing. Accordingly, the city of Seoul is currently increasing the number of central bus-only lanes designed to allow buses to run smoothly within the city center through one inner lane. According to the ‘Statistics on Seoul City Bus Stops’ recently surveyed by the Seoul Metropolitan Government, the number of central bus stops has increased approximately fourfold from 98 in 2004 to 400 in 2023 [1]. In Korea, most roads except for highways use asphalt concrete pavement, which is a flexible pavement. The central bus stops in Seoul are also constructed with asphalt concrete pavement, and as a result of frequent acceleration and deceleration of heavy vehicles such as buses and bad weather, the asphalt concrete pavement is deteriorating rapidly, resulting in an increase in pavement damages such as cracks, rutting, and potholes, as shown in Figure 1. The damages to asphalt concrete pavement cause inconvenience to users and require frequent maintenance, resulting in significant costs.

**Figure 2:**
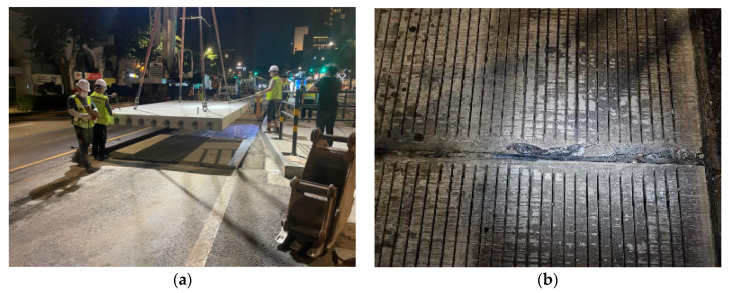
Cement concrete pavement: (**a**) precast concrete pavement; (**b**) joint damage.

Internationally, there are cases where cement concrete pavement, a type of rigid pavement, is used to ensure the durability of bus stop pavement. Several states in the United States, including New York, Texas, California, Ohio, Maryland, and Delaware, are implementing cement concrete pavement for downtown bus stops [2,3,4,5,6,7,8,9]. In addition, many countries, including Canada [10], the United Kingdom [11], Sweden [12], Taiwan [13], and Ethiopia [14], are using cement concrete pavement at bus stops in urban areas. Recently, the city of Seoul has been applying the precast concrete pavement method, as shown in Figure 2, to convert the asphalt concrete pavement in use in the central bus stop section, where frequent damage occurs, to a rigid pavement. The precast concrete pavement method can be used to rapidly convert asphalt concrete pavement into cement concrete pavement by using concrete slabs prefabricated in a factory. Several countries are trying to improve the performance of precast concrete pavement, including the United States [15,16,17,18], China [19,20,21,22], Spain [23], the Czech Republic [24,25] and India [26,27]. However, because the precast concrete pavement method is a high-cost method, its widespread application is limited. Therefore, low-cost, cast-in-place cement concrete pavement can be applied to the pavement of newly constructed central bus stops that are not currently in use.

Most cement concrete pavements constructed at bus stops are the jointed concrete pavements (JCPs). The JCP is the most widely used cement concrete pavement type, but it has problems such as frequent joint damage, as shown in Figure 2b, and reduced smoothness due to curling of JCP slabs [28,29,30,31,32]. A more durable cement concrete pavement type is the continuously reinforced concrete pavement (CRCP). In the CRCP, the transverse contraction joints are not required, and instead transverse cracks are permitted, with the steel bars placed continuously in the longitudinal direction holding the cracks tightly. Many studies have been conducted to improve the performance of CRCP, with major research topics including behavior analysis [33,34,35,36,37], materials [38,39,40], and design [41,42,43,44]. Recently, the city of Seoul constructed a new central bus stop pavement using CRCP. Since this is the first time that cast-in-place CRCP has been applied to the central bus stop pavement in Korea and there are limited cases of applying CRCP to the bus stop pavement [45,46,47], an analysis of its usability is necessary.

The purpose of this study is to analyze the behavior and performance of the cast-in-place CRCP applied to bus stop pavement in a central bus-only lane. Through this study, the appropriateness of the central bus stop CRCP (hereinafter referred to as the CRC bus pad) can be evaluated, and the results can be used as data for optimizing the application of the CRC bus pad in the future. In addition, the reliability of the application of the CRC bus pad is intended to be increased. To achieve these goals, a plan for analyzing the behavior and performance of the CRC bus pad was first established, and various behaviors of the concrete slab and the continuous steel bars of the CRC bus pad in response to environmental loads were measured and analyzed, and the stresses in the concrete slab and steel bars in response to the loads of a moving vehicle were identified to evaluate the performance along with the finite element analysis. This paper describes the research contents and results in detail.

## 2. CRC Bus Pad Construction and Experimental Plan

### 2.1. Design and Construction of CRC Bus Pad

In order to evaluate the behavior and performance of the CRC bus pad in the central bus stop section, which is the most frequently damaged section of the central bus-only lane, a CRC bus pad was constructed at one of the central bus stops in Seoul in June 2024. The width of the CRC bus pad was designed to be 2.9 m, which is the lane width, and the overall length was designed to be 70 m to include the sections where vehicles decelerate when entering a bus stop and the section where they accelerate when exiting a bus stop. The thicknesses of the pavement layers and the longitudinal reinforcement ratio were designed by applying the standard design of highway CRCP, with the slab thickness being 0.3 m, the longitudinal reinforcement ratio being 0.68%, and the lean concrete subbase thickness being 0.15 m [48,49]. Since the pavement other than the CRC bus pad is asphalt concrete pavement, 3 m long tapered approach slabs were applied to the sections where the CRC bus pad and asphalt pavement are connected to alleviate the sudden change in pavement stiffness. The CRC bus pad and approach slabs were connected with dowel bars to facilitate load transfer.

**Figure 3:**
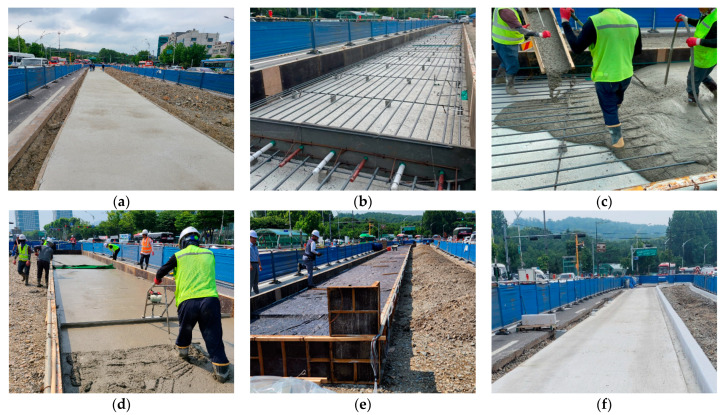
Major construction processes of cast-in-place CRC bus pad: (**a**) lean concrete subbase placement; (**b**) formwork installation and steel bar placement; (**c**) cement concrete pouring work; (**d**) surface smoothing work; (**e**) curing blanket covering work; (**f**) CRC bus pad completion.

The major construction processes of the cast-in-place CRC bus pad are shown in Figure 3. First, the existing asphalt concrete pavement is removed, and a lean concrete subbase is constructed. Then, the formwork is installed and the longitudinal and transverse steel bars are placed. Next, the concrete is poured, compacted using a hand vibrator, and leveled using a hand screed. After leveling the pavement surface, fine roughness of the pavement surface is created using carpet drag, and curing is performed by covering it with a curing blanket. After curing is completed, the curing blanket is removed, and grooving work is performed to form grooves on the pavement surface to reduce hydroplaning and improve skid resistance.

### 2.2. Experimental Plan

**Figure 4:**
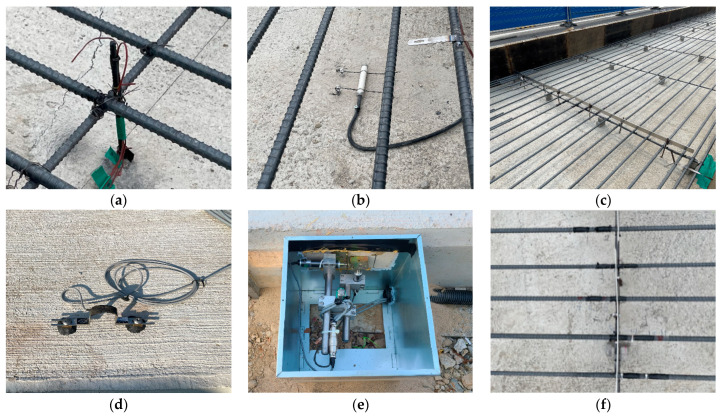
Preparation for measurement: (**a**) temperature sensors; (**b**) concrete strain gauge; (**c**) crack inducer; (**d**) crack gauge; (**e**) displacement transducers; (**f**) steel strain gauges.

**Figure 5:**
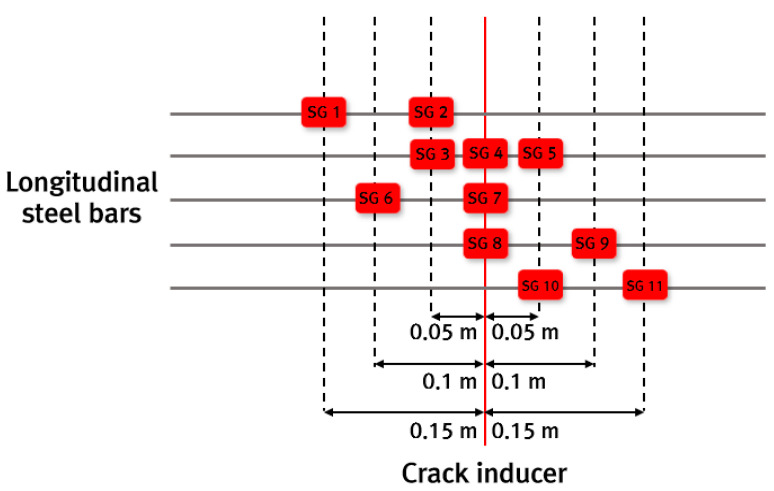
Locations and gauge numbers of steel strain gauges.

**Figure 6:**
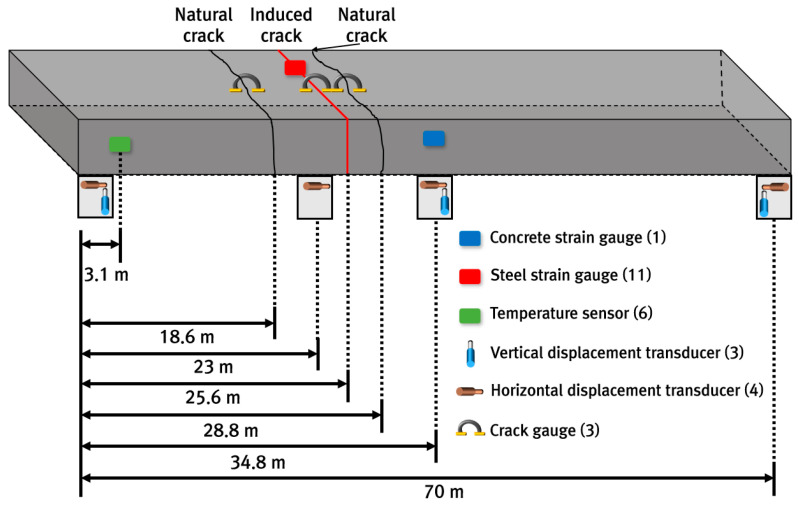
Summary of measurement gauges and locations.

To analyze the behavior of the CRC bus pad, measurements were performed by installing temperature sensors, concrete strain gauges, crack inducers, steel strain gauges, crack gauges, and displacement transducers. As shown in Figure 4a, temperature sensors were installed at the bottom (0 m), middle (0.15 m), and top (0.29 m) of the slab and in the air to analyze temperature changes, which are environmental loads, according to the depth of the slab. A total of six temperature sensors were installed: one in the air, two at the top, one at the middle depth, and two at the bottom of the slab. To measure the strain of the CRC bus pad under environmental and moving vehicle loads, a concrete strain gauge was installed at the bottom (0.02 m) of the slab in the wheel pass area at the center of the bus pad where frequent acceleration and deceleration of buses occur, as shown in Figure 4b. The concrete strain gauge was installed at the bottom of the slab to measure the maximum strain due to bending of the slab, and the longitudinal steel bars were placed at the middle depth of the slab so they do not interfere with each other. The concrete strain gauge used in this experiment was KM-100B (manufactured by TML, Tokyo, Japan), which has a strain capacity of ±5000 × 10^−6^, gauge length of 100 mm, gauge diameter of 17 mm, and allowable temperature range of −20 °C to 80 °C. As shown in Figure 4c, a crack inducer was made by cutting a stainless steel sheet, and after fixing the supporting steel bars vertically, the crack inducer was firmly tied to them to prevent it from moving when pouring concrete. At the location where the crack inducer is installed, the cross-sectional area of the concrete slab decreases, causing stress concentration and, as a result, a crack to occur. A crack inducer was installed at a location 25.6 m from the entry location of the CRC bus pad to analyze the stress characteristics of the steel bars due to crack occurrence in the CRC bus pad. A total of three crack gauges were installed on natural and induced cracks, as shown in Figure 4d, to analyze crack width behavior, which is closely related to the performance of the CRC bus pad. As shown in Figure 4e, a total of three vertical displacement transducers were installed at the entry, center, and exit locations of the CRC bus pad to analyze the curling behavior of the CRC bus pad, and a total of four horizontal displacement transducers were installed at several different longitudinal locations on the side of the CRC bus pad to analyze the longitudinal behavior by location. These measurement sensors were protected with enclosures to prevent damage from rainwater. In order to analyze the steel bar stresses in the crack area where the large stresses of the steel bars occur in the CRC bus pad, a total of eleven steel strain gauges were installed as shown in Figure 4f. Details of the locations and gauge numbers of the steel strain gauges are shown in Figure 5. Since a crack does not occur in a perfectly straight line and there is some deviation even when a crack is induced using a crack induction device, multiple steel strain gauges were attached to several steel bars around the crack location to identify the steel bar strains in the crack region. Since insulating tape is used when attaching steel strain gauges, if the gauge spacing is too narrow or too many gauges are attached to one steel bar, the bond between the steel bar and concrete will decrease, and the measured strains may be different from the actual steel bar strains of the CRCP. Therefore, the minimum spacing between gauges was set to 0.05 m, and a maximum of three gauges were attached to one steel bar. The steel strain gauge numbers are assigned sequentially starting from the left gauge of the outermost steel bar to the right gauge of the innermost steel bar. The steel strain gauge used in this experiment was FLA-6 (manufactured by TML, Tokyo, Japan), which has a strain capacity of ±50,000 × 10^−6^, gauge length of 6 mm, gauge width of 2.2 mm, and allowable temperature range of −20 °C to 80 °C. The overall measurement plan for analyzing the behavior of the CRC bus pad is summarized in Figure 6.

## 3. Behavior of CRC Bus Pad Under Environmental Loads

The behavior of the CRC bus pad subjected to environmental loads was comprehensively analyzed in this study since the early-age performance of CRCP is known to be mainly affected by environmental loads [50,51,52]. First, the temperature variation in the concrete slab of the CRC bus pad was analyzed by measuring the temperature change in the slab. Changes in air temperature cause temperature changes in the concrete slab, which in turn cause expansion, contraction, and curling of the slab. These behaviors cause stresses in the concrete slab and steel bars of the CRC bus pad. The deformation characteristics of the concrete slab and the steel bars inside the CRC bus pad were identified by analyzing the measured strains of the concrete slab and the steel bars. The change in crack width was investigated by measuring the amount of crack opening and closing. In addition, the curling behavior of the CRC bus pad was analyzed by measuring the vertical displacements, and the movement of both ends of the CRC bus pad was also analyzed by measuring the horizontal displacements.

### 3.1. Temperature Variation in Concrete Slab

**Figure 7:**
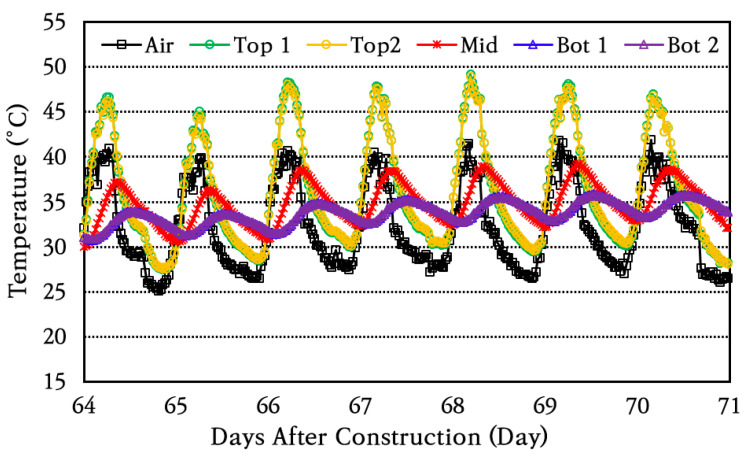
Temperature variation in air and through slab depth.

After construction, the temperature changes at the top, middle, and bottom of the concrete slab where temperature sensors were installed and in the air were measured and analyzed. As an example, the results of analyzing the temperature changes over a week are shown in Figure 7. As can be seen in the figure, the temperature at the top of the slab first changes in a similar trend as the air temperature changes, and the amount of temperature change decreases from the top to the bottom of the slab. It can also be seen that the time at which the maximum or minimum temperature occurs is delayed from the top to the bottom of the slab. It is noted that changes in concrete slab temperature are also affected by the heat of hydration of the concrete in addition to the air temperature changes, especially during the initial few days.

**Figure 8:**
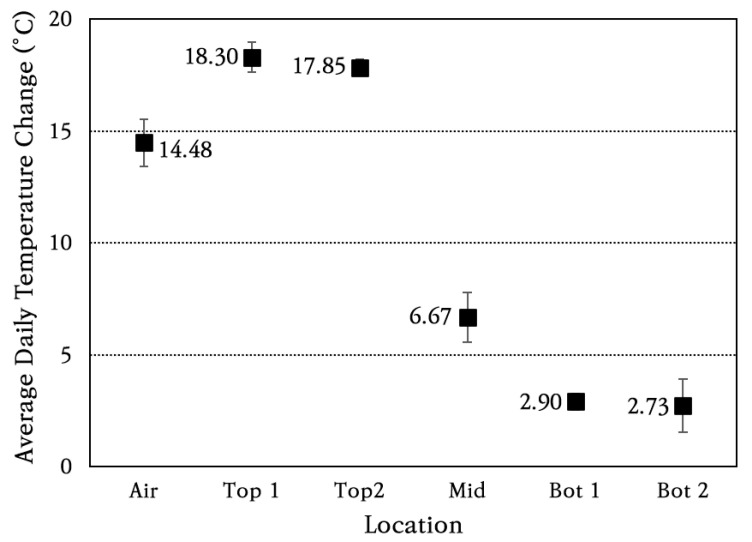
Average daily temperature changes in air and through slab depth.

For a more detailed analysis, the daily temperature change was analyzed by slab depth, and the average and standard deviation of daily temperature change are shown in Figure 8. The coefficient of variation of daily temperature change is found to be within approximately 10%. The average daily temperature change is greatest at the top of the slab, decreasing toward the atmosphere, the middle of the slab, and the bottom of the slab. The temperature change at the top of the slab is larger than the air temperature change because the temperature of the concrete slab surface rises higher than the air temperature due to direct sunlight during the day. In addition, the temperature difference between the top and bottom of the slab is large, approximately 15 °C, and the temperature change becomes significantly smaller as the depth of the slab increases. The temperature change patterns of the concrete slab analyzed in this study are similar to those reported in several previous studies [36,53,54].

### 3.2. Strain of Concrete Slab

**Figure 9:**
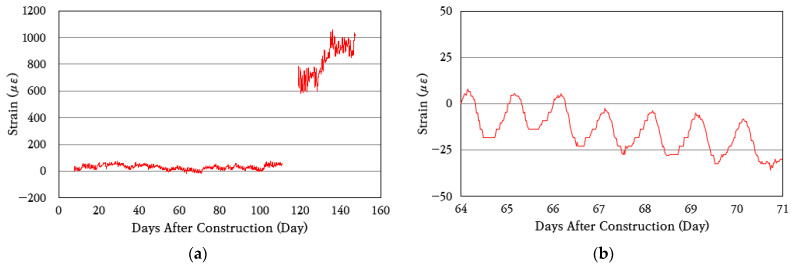
Concrete strain variation: (**a**) total period; (**b**) one-week period.

To analyze the behavior characteristics of the CRC bus pad, the strain of the concrete slab was measured and analyzed using a concrete strain gauge installed at the central area of the bus pad. Figure 9a shows the change in concrete strain from the day of construction, and Figure 9b shows the change in strain over a random week among them for an enlarged view. For approximately 110 to 120 days after construction, measurements could not be properly performed due to poor battery connection in the datalogger. As can be seen in the figure, the concrete strain periodically increases and decreases according to the temperature change. The average daily strain change of the concrete slab is approximately 25 με, which indicates that very small strain occurs. However, approximately 120 days after construction, a crack occurred at the location of the concrete strain gauge, and as a result, the concrete strain measurement values increased significantly. It was analyzed that this was because, after the crack occurred, the strain gauge measured the displacements according to the opening and closing behavior of the crack rather than the strains of the concrete slab, and thus the measured values increased.

### 3.3. Strain of Steel Bar

**Figure 10:**
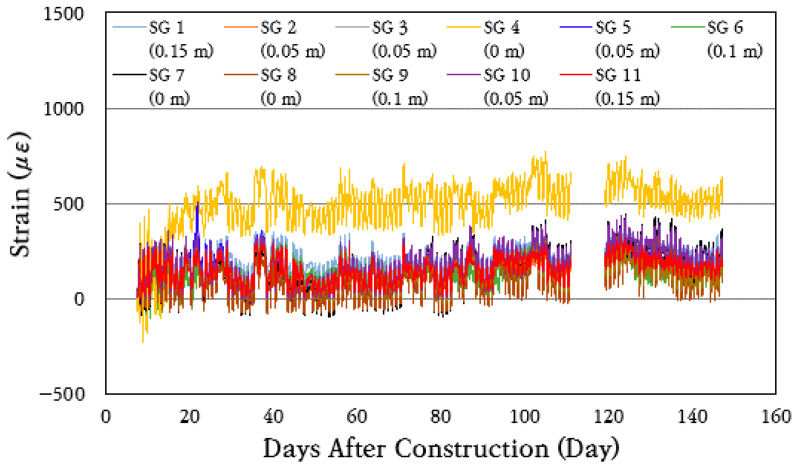
Steel strain variation throughout total period.

To analyze the behavior characteristics of the steel bars in the CRC bus pad, the strains of the steel bars in the cracked area where large stresses occur in the steel bars were measured and analyzed using the steel strain gauges installed at 0 m (SG 4, SG 7, SG 8), 0.05 m (SG 2, SG 3, SG 5, SG 10), 0.10 m (SG 6, SG 9), and 0.15 m (SG 1, SG 11) distances from the induced crack, and the results are shown in Figure 10. For approximately 110 to 120 days after construction, measurements could not be properly performed due to poor battery connection in the datalogger. As can be seen in the figure, the steel strains periodically increase and decrease like the concrete strain.

**Figure 11:**
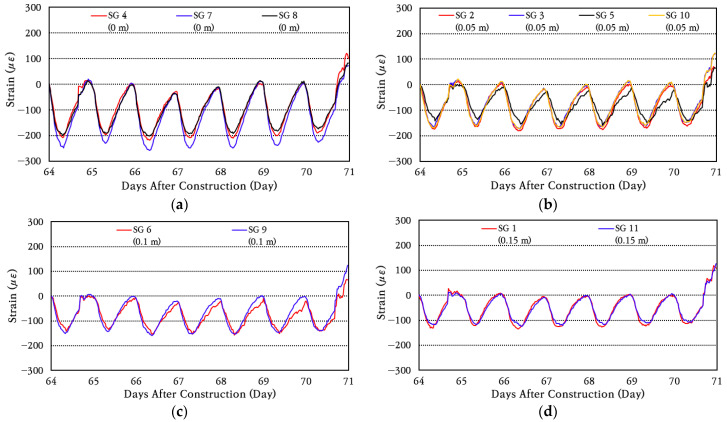
Steel strain variation at: (**a**) crack location; (**b**) 0.05 m from crack; (**c**) 0.10 m from crack; (**d**) 0.15 m from crack.

To analyze the behavior of longitudinal steel bars in more detail, the strain data for a random week were analyzed by strain gauges installed at the same distance from the crack and are shown in Figure 11. As can be seen in the figure, the strains of steel bars change similarly when the distance from the crack is the same. The largest strain changes occur in the strain gauges installed at the crack, and the smallest strain changes occur in the strain gauges installed 0.15 m away from the crack. Therefore, it is confirmed that the maximum strain and strain variation range of the steel bars decrease as the distance from the crack increases. It can be seen that the steel bar strain increases rather sharply on day 71, which is instantaneous, as can be seen in Figure 10, and is caused by a more rapid decrease in the temperature of the slab, as can be seen in Figure 7. As the temperature of the slab decreases, the slab contracts, increasing the steel bar strain around the crack.

**Figure 12:**
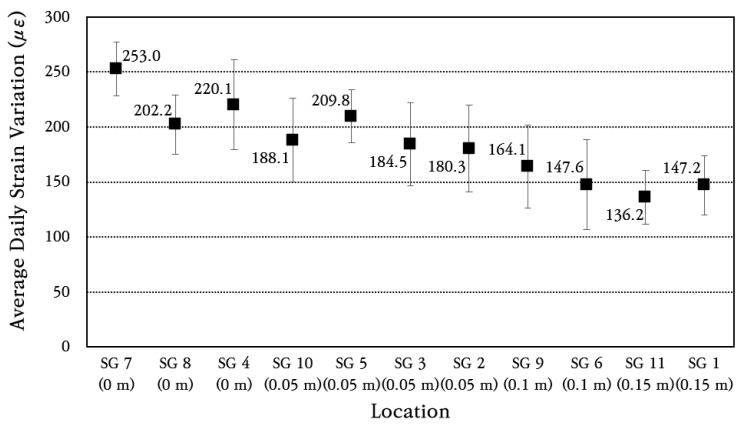
Average daily steel strain variation at different distances from crack.

In order to analyze the strain change pattern of longitudinal steel bars in more detail, the range of strain change for each day is calculated, and then the average and standard deviation of daily strain change are obtained and shown in Figure 12. The coefficient of variation of daily strain change is found to be about 20%. Comparing the maximum value of the average daily strain change in the strain gauge installed at the induced crack with the maximum value of the average daily strain change in the strain gauge installed at a location 0.05 m away from the crack, it can be seen that the strain change is reduced by about 17% at a location 0.05 m away. It is analyzed that the maximum value of the change in the strain of the steel bar at a location 0.10 m away from the crack is reduced by approximately 35%, and the maximum value of the change in the strain of the steel bar at a location 0.15 m away is reduced by approximately 42%. This analysis results show that the daily strain change in the steel bar occurs greatest at the crack location and that the strain change in the steel bar decreases significantly as it moves away from the crack. In particular, the strain of the steel bar decreases significantly even when moving away from the crack by only about 0.15 m, which means that the bond between concrete and steel bars is excellent. In the CRCP, the steel bars take all the stress at the crack, and as one moves away from the crack, the steel bar stress decreases and the concrete stress increases due to the bond between the concrete and steel bars. If the bond between concrete and steel bars is excellent, the steel bar stress will be significantly reduced even if the distance from the crack is small. On the other hand, if the bond is poor, the steel bar stress will not be significantly reduced even if the distance from the crack is large. In addition, if the strain of the steel bar that occurred at the crack location is converted to the stress of the steel bar by applying the elastic modulus of the steel bar of 200 GPa, it is approximately 50 MPa. Since this is significantly smaller than the yield strength of 400 MPa of the steel bar, the range of stress acting on the steel bar is analyzed to be appropriate for ensuring excellent performance of the CRC bus pad.

### 3.4. Crack Width Behavior

**Figure 13:**
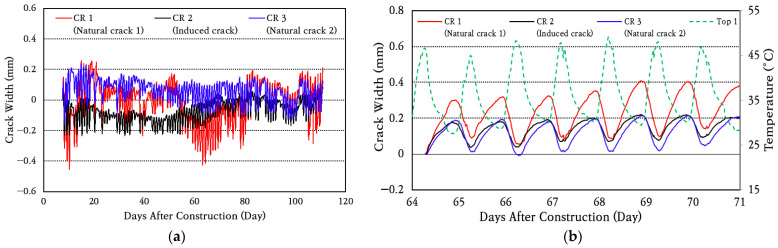
Crack width variation: (**a**) total period; (**b**) one-week period.

In order to analyze the crack width behavior, which is a major performance index of the CRC bus pad, data collected from a total of three crack gauges installed at two locations where natural cracks (CR 1 and CR 3) occurred and one location where an induced crack (CR 2) occurred were analyzed, and the results are shown in Figure 13. To analyze the crack width behavior in more detail, the crack width behavior for a random week is shown in Figure 13b. It can be seen that the cracks are continuously opening and closing every day depending on the temperature change. As the temperature rises, the concrete slab expands, decreasing the crack width. Conversely, as the temperature drops, the slab contracts, increasing the crack width.

**Figure 14:**
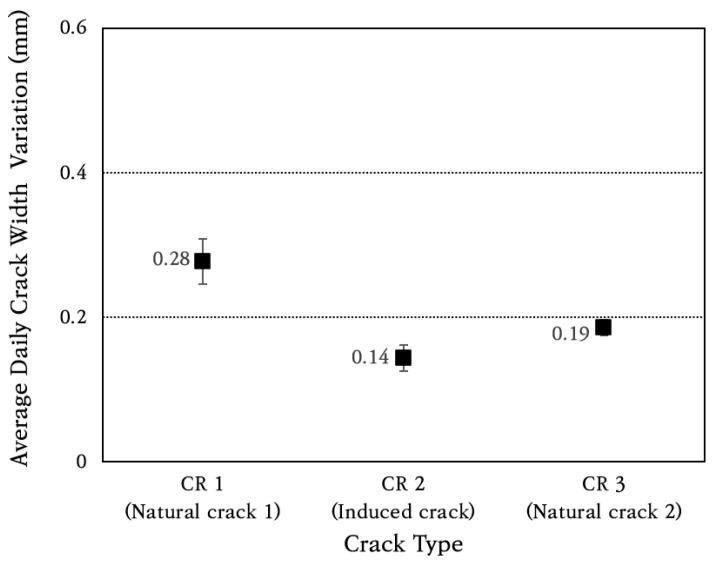
Average daily crack width variation.

The average and standard deviation of daily crack width change were calculated, and the results are shown in Figure 14. The coefficient of variation of daily crack width change is found to be about 10%. The average daily crack width change is analyzed to be approximately 0.28 mm in natural crack 1 (CR 1), which is the largest among the three cracks, and the crack width change in the induced crack (CR 2) is found to be the smallest at approximately 0.14 mm. It is confirmed that the amount of crack width change is very small, and, therefore, it does not cause a decrease in the performance of the CRC bus pad.

### 3.5. Vertical Displacement of Concrete Slab

**Figure 15:**
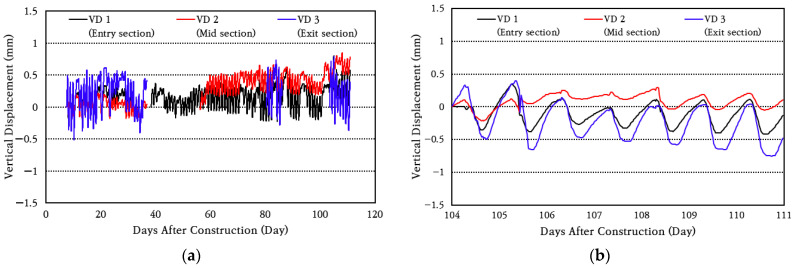
Vertical displacement variation: (**a**) total period; (**b**) one-week period.

In order to identify the curling phenomenon, which is a change in vertical displacement of the CRC bus pad, the data from the vertical displacement transducers installed at the entry (VD 1), center (VD 2), and exit (VD 3) sections of the bus pad were analyzed, and the results are shown in Figure 15. It is noted that each displacement transducer suffered flooding damage due to frequent heavy rain during the measurement period, and the data from that period was deleted from the analysis. The flood-damaged displacement transducers were replaced with new ones, and measurements were performed continuously. As can be seen in the figure, during the day, the temperature at the top of the slab is higher than that at the bottom, so the top of the slab expands more, causing the slab to bend downward (curl-down), which reduces the vertical displacement on the side of the slab. Conversely, during the night, the top of the slab contracts more than the bottom of the slab, causing a curl-up phenomenon, which increases the vertical displacement on the side of the slab.

**Figure 16:**
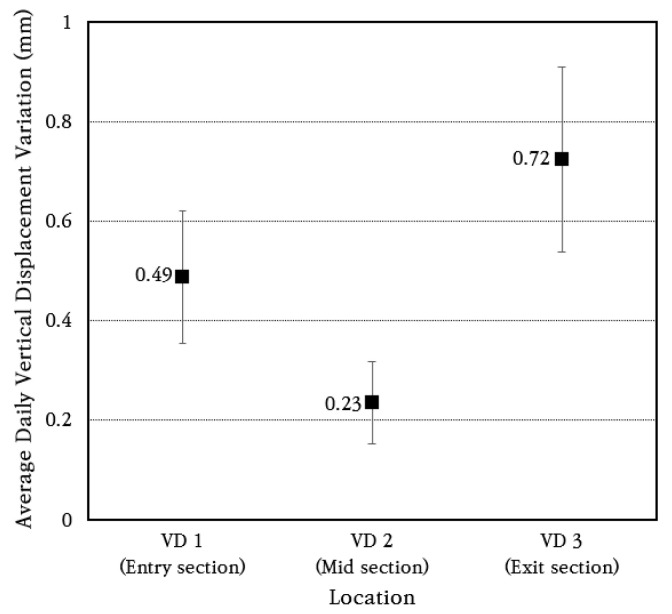
Average daily vertical displacement variation.

To analyze the vertical displacement characteristics of the CRC bus pad in more detail, the vertical displacement data for a random week was analyzed to obtain the average and standard deviation of daily vertical displacement change, which are shown in Figure 16. The coefficient of variation of daily vertical displacement change is found to be about 30%. The average daily vertical displacement changes were 0.49 mm at the bus pad entry section (VD 1), 0.23 mm, at the smallest, at the central section (VD 2), and 0.72 mm, the largest, at the exit section (VD 3). Therefore, it can be seen that the vertical displacement of the CRC bus pad occurs the largest at the entry and exit sections at both ends, and the vertical displacement decreases as it moves toward the center. However, since the magnitude of this vertical displacement change is very small despite the CRC bus pad being quite long at 70 m, it is evaluated that the curling phenomenon of the CRC bus pad will not have a negative effect on pavement performance.

### 3.6. Horizontal Displacement of Concrete Slab

**Figure 17:**
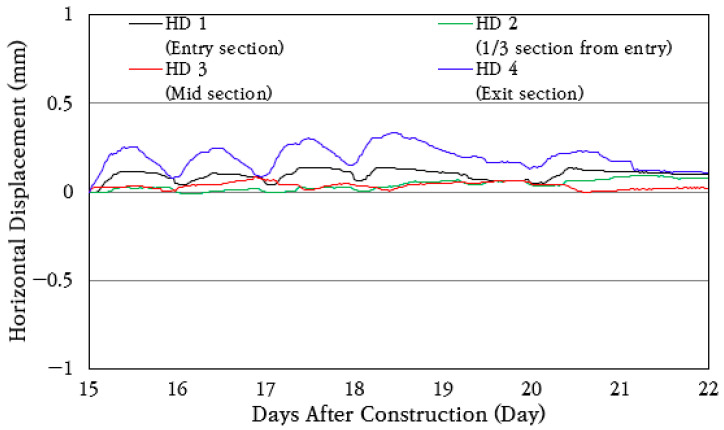
Horizontal displacement variation.

To analyze the longitudinal behavior of the CRC bus pad, horizontal displacement transducers were installed and measured at the entry section (HD 1), the 1/3 section of the extension (HD 2), the central section (HD 3), and the exit section (HD 4) of the bus pad, and the horizontal displacement change over a week is shown in Figure 17. As shown in the figure, the bus pad experiences repeated contraction and expansion every day depending on temperature changes. During the day, the temperature rises and the bus pad expands, and during the night, the temperature drops and the bus pad contracts.

**Figure 18:**
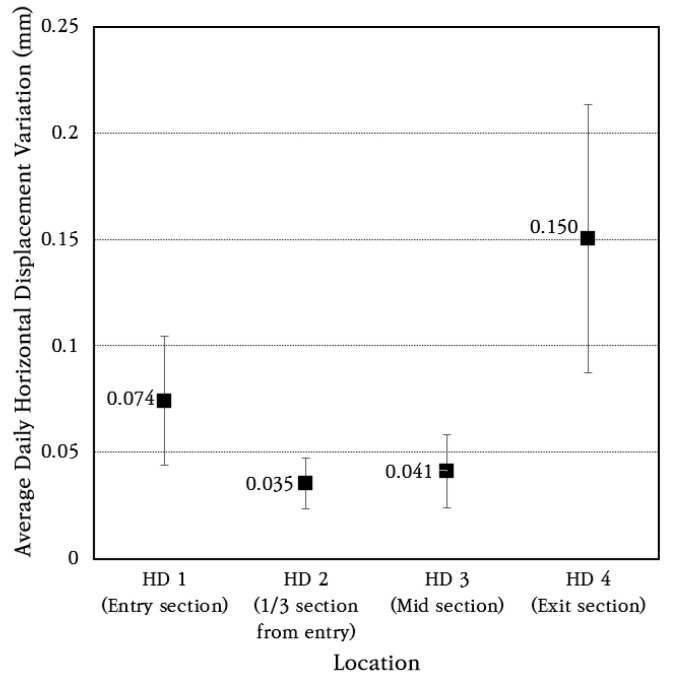
Average daily horizontal displacement variation.

The average and standard deviation of daily horizontal displacement change were analyzed, and the results are shown in Figure 18. The coefficient of variation of daily horizontal displacement change is found to be about 40%. The longitudinal displacements of 0.074 mm and 0.15 mm occurred at the bus pad entry section (HD 1) and exit section (HD 4), respectively, and smaller longitudinal displacements of 0.035 mm and 0.041 mm occurred at the 1/3 section of the extension (HD 2) and the central section (HD 3), respectively. Therefore, even if the CRC bus pad is long, very small longitudinal displacements occur at the entry and exit sections due to frictional resistance with the subbase layer, and the longitudinal displacements are small enough to be ignored in inner sections.

## 4. Behavior of CRC Bus Pad Under Moving Vehicle Loads

To analyze the dynamic behavior of the concrete slab and longitudinal steel bars of the CRC bus pad due to moving vehicles, a dynamic moving load test was conducted using a dump truck. The load of the vehicle was 132 kN on an empty vehicle weight of 113 kN, making a total of 245 kN as the experimental load. The front axle of the vehicle is a single-wheel single axle, and the rear axle is a dual-wheel tandem axle. The load ratio of the front axle is 21%, which is 51.5 kN; the load ratio of the front axle of the tandem axle is 49%, which is 120 kN; and the load ratio of the rear axle of the tandem axle is 30%, which is 73.5 kN. It is noted that the experiment was conducted before the bus pad opened and only construction vehicles were allowed to pass during construction. Therefore, a bus could not be used as an experimental vehicle, and a dump truck that was heavier than a bus was used as an experimental vehicle for conservative analysis. For reference, the weight of an empty bus is about 103 kN, and the gross weight is about 162 kN.

**Figure 19:**
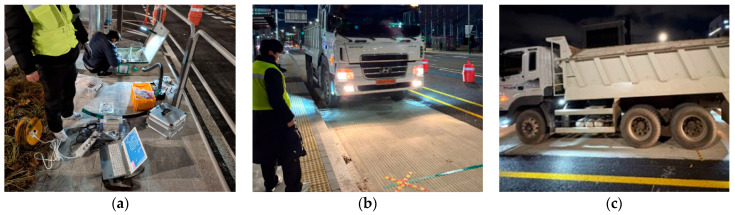
Dynamic load test using moving vehicle: (**a**) data logging system; (**b**) vehicle moving on bus pad; (**c**) vehicle stopping on bus pad.

The experiments were conducted in five ways, as shown in Table 1. The first method for measuring the strain of the concrete slab is to simulate a situation in which a vehicle passes the bus pad without stopping, with the vehicle moving at a constant velocity and the right wheels passing the gauge location. The second method is to simulate a situation in which a vehicle stops on the bus pad and then starts moving again, with the right front wheel of the tandem axle temporarily stopping at the gauge location. The third method is to simulate the wandering situation of a moving vehicle by having the vehicle move at a constant velocity about 0.3 m away transversely from the gauge location. The strain measurement methods of longitudinal steel bars are as follows: first, the vehicle is moving at a constant velocity, and the right wheels pass the gauge location; second, the vehicle’s right front wheel of the tandem axle stops at the gauge location and then starts moving again. The experiments were conducted three times each, and Figure 19 shows the dynamic measurement system and the experimental scene. In this study, since the cases where a bus stopped or passed slowly after entering a bus stop were considered, experiments were conducted at vehicle velocities of up to 40 km/h. When conducting experiments, it seemed that this velocity was quite high at the bus stop.

### 4.1. Dynamic Strain Analysis of Concrete Slab

**Figure 20:**
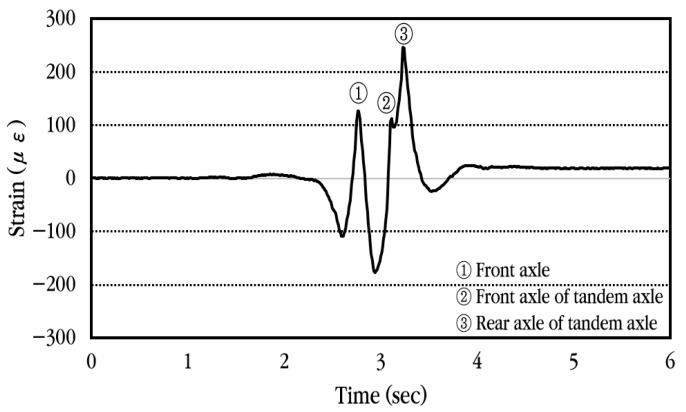
Dynamic concrete slab strain history under constant vehicle velocity.

To analyze the dynamic response of the concrete slab of the CRC bus pad subjected to moving vehicles, experimental data were collected using a concrete strain gauge installed at the center of the bus pad. First, the dynamic strain history occurring in the slab when a vehicle passes the bus pad at a constant velocity without stopping is analyzed and shown in Figure 20. The concrete strain is measured as positive when tension occurs at the bottom of the slab and as negative strain when compression occurs. As can be seen in the figure, when the front axle of the vehicle approaches the strain gauge location, compressive strain occurs first at the bottom of the slab, and when the front axle is positioned at the strain gauge location, tensile strain occurs. Afterwards, when the vehicle moves and the tandem axle approaches the strain gauge location, compressive strain occurs again, and when the front axle of the tandem axle is positioned at the strain gauge location, tensile strain occurs, and when the rear axle of the tandem axle is positioned at the strain gauge location, tensile strain becomes maximum. Eventually, when the tandem axle passes through the strain gauge, compressive strain occurs again at the bottom of the slab, and then the strain disappears. The corresponding strains are also labeled in the figure when the front axle, the front axle of the tandem axle, and the rear axle of the tandem axle are placed at the gauge position.

**Figure 21:**
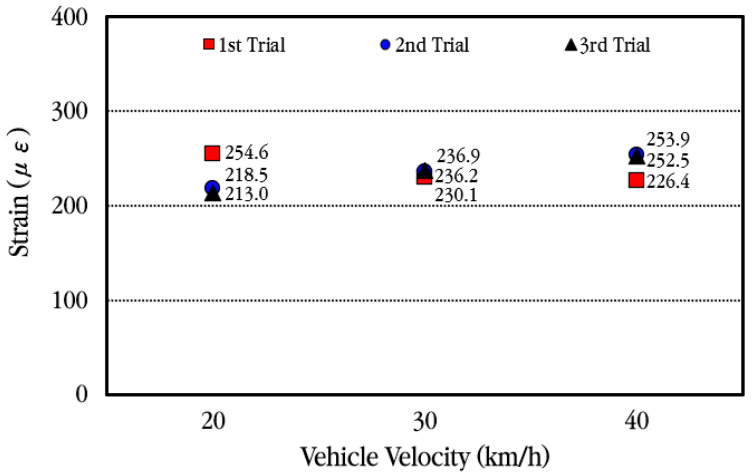
Maximum concrete slab strains depending on vehicle velocity.

The maximum strain of the slab according to the vehicle velocity when the vehicle passes through the bus pad at a constant velocity is analyzed, and the results are shown in Figure 21. Considering that the CRC bus pad is a bus stop, the experiment was conducted at low vehicle velocities of 20 km/h, 30 km/h, and 40 km/h. As previously explained, the maximum strain occurs when the tandem axle passes through, and the measured values show slight differences depending on each experiment. Overall, it can be seen that the maximum strain occurs in a similar range depending on the change in vehicle velocity. Therefore, it is analyzed that the strain of the slab when a vehicle passes through the CRC bus pad has almost no relation to the vehicle velocity within the vehicle velocity range used in this experiment. It is also noted that a one-way ANOVA was performed, and the result revealed that there was no statistically significant difference in the relationship between slab strain and vehicle velocity.

**Figure 22:**
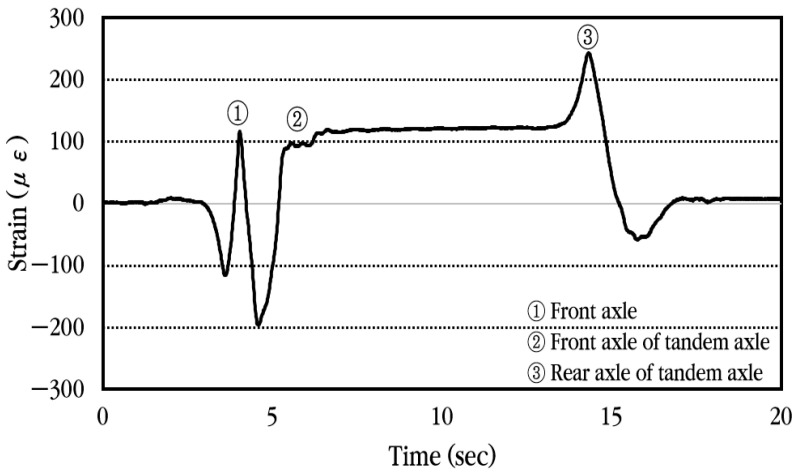
Dynamic concrete slab strain history when vehicle is moving and stopping.

Next, the dynamic strain history occurring in the slab is analyzed and shown in Figure 22 when the vehicle gradually decelerates upon entering the bus stop and stops so that the front axle of the tandem axle is positioned at the concrete strain gauge location, then starts moving again. As in the previous case, when the front axle of the vehicle approaches the strain gauge location, compressive strain occurs first at the bottom of the slab, and when the front axle is positioned at the strain gauge location, tensile strain occurs. Afterwards, when the tandem axle approaches the strain gauge location, compressive strain occurs again, and when the front axle of the tandem axle is positioned at the strain gauge location, tensile strain occurs. When the vehicle moves again and the rear axle of the tandem axle is positioned at the strain gauge location, tensile strain becomes maximum. Finally, when the tandem axle passes through the strain gauge, compressive strain occurs again at the bottom of the slab, and then the strain becomes zero.

**Figure 23:**
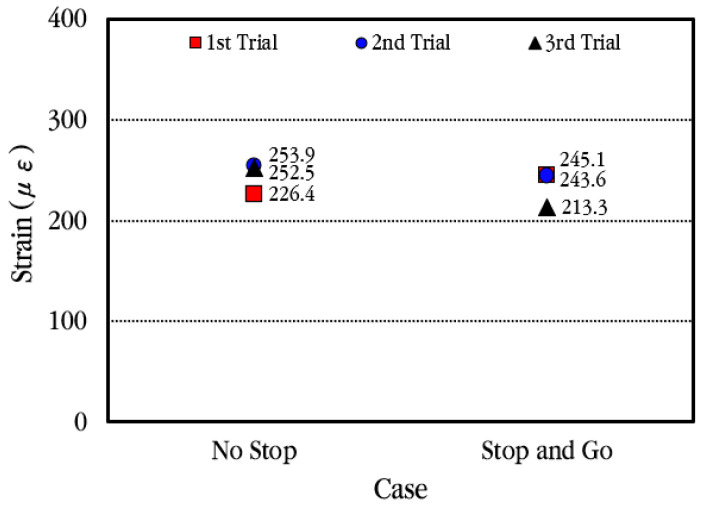
Maximum concrete slab strains depending on vehicle movement case.

Figure 23 compares the maximum strain of the slab when the vehicle stops at the concrete strain gauge location temporarily and when the vehicle moves without stopping. As can be seen in the figure, although there is a slight difference in the measured values depending on each experiment, in general, the maximum strain occurs similarly regardless of whether the vehicle stops. Therefore, it is analyzed that when a vehicle passes through the CRC bus pad, the strain of the slab is hardly affected by the acceleration and deceleration of the vehicle.

**Figure 24:**
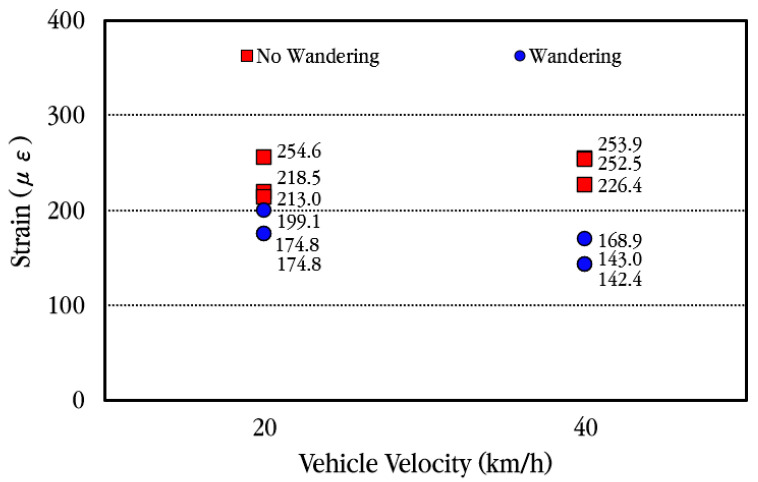
Maximum concrete slab strains depending on transverse wandering of vehicle.

When a vehicle passes the CRC bus pad, in order to analyze how much the slab strain decreases when the wheels are spaced transversely from the location of the concrete strain gauge, the data were collected and analyzed by having the vehicle move at a location approximately 0.3 m away transversely from the concrete strain gauge, and the results of the analysis are shown in Figure 24. As shown in the figure, when the vehicle moves away transversely from the strain gauge, the maximum strain decreases regardless of the vehicle velocity. In the figure, the wandering effect is shown to be larger when the vehicle speed is 40 km/h than when it is 20 km/h. This is believed to be due to the vehicle moving further away from the strain gauge rather than the effect of the vehicle velocity. Therefore, it is analyzed that when a vehicle moves with a slight transverse wandering in the wheel pass section where the strain gauge is installed, the strain in the wheel pass section is significantly reduced.

### 4.2. Dynamic Strain Analysis of Steel Bar

To analyze the dynamic response of the longitudinal steel bars of the CRC bus pad subjected to moving vehicles, experimental data were collected using the steel strain gauges installed in the cracked area where the steel bar stresses are large. Since the steel strain gauges are installed on the top of the steel bars, the steel bar strain is measured as positive when tension occurs on the top of the steel bar and as negative when compression occurs. In addition, since the longitudinal steel bars are placed at the middle depth of the slab, which is the neutral axis of the slab, the strains that occur at the top and bottom of the steel bar due to moving vehicles are the same in magnitude, although the directions are opposite.

**Figure 25:**
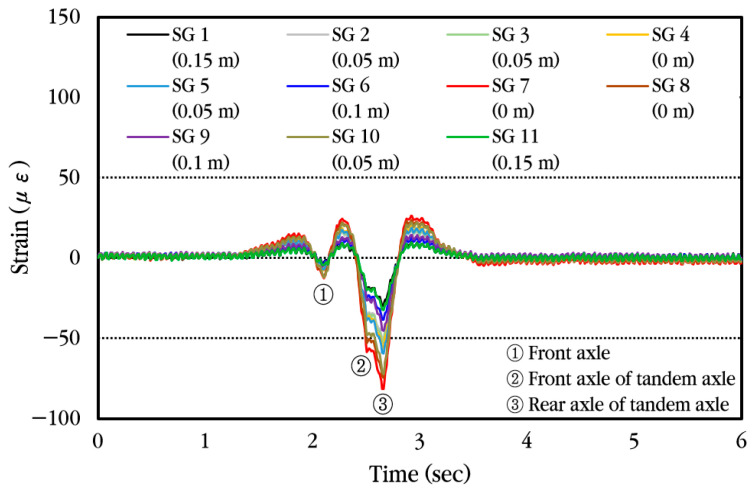
Dynamic steel bar strain history under constant vehicle velocity.

First, the dynamic strain histories occurring in different locations of the steel bars when a vehicle passes the bus pad at a constant velocity without stopping are analyzed and shown in Figure 25. As can be seen in the figure, the pattern of change in strain is similar regardless of the installation location of the strain gauge, but the magnitude of the strain is different. When the front axle of the vehicle approaches the strain gauge location, tensile strain occurs first at the top of the steel bar, and when the front axle is positioned at the strain gauge location, the compressive strain occurs. Afterwards, when the vehicle moves and the tandem axle approaches the strain gauge location, tensile strain occurs again, and when the front axle of the tandem axle is positioned at the strain gauge location, compressive strain occurs, and when the rear axle of the tandem axle is positioned at the strain gauge location, compressive strain becomes maximum. Eventually, when the tandem axle passes through the strain gauge, tensile strain occurs again at the top of the steel bar, and then the strain disappears.

**Figure 26:**
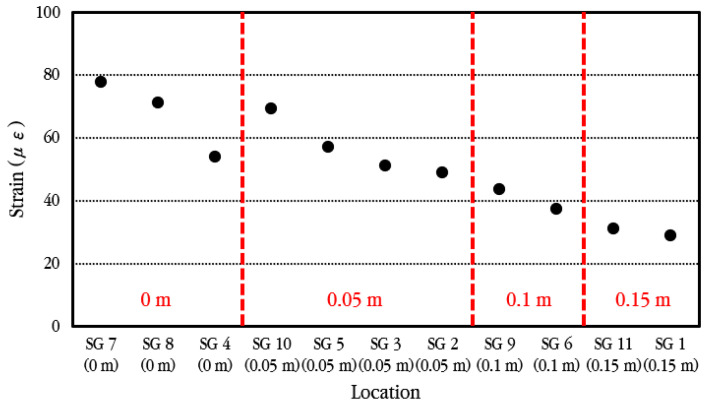
Maximum dynamic steel bar strains depending on distances from crack.

The maximum strain of longitudinal steel bars according to the measurement location of steel bar strain is analyzed and shown in Figure 26. As shown in the figure, the strain of the steel bar is largest at the crack location and decreases as it moves away from the crack. Additionally, even at the same distance from the crack, the strain occurs larger in the steel bar through which the wheel passes closer.

**Figure 27:**
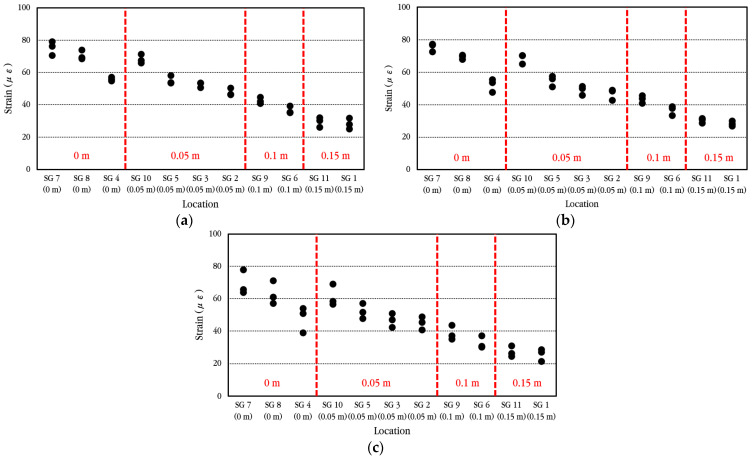
Maximum dynamic steel bar strains depending on vehicle velocity of (**a**) 20 km/h; (**b**) 30 km/h; (**c**) 40 km/h.

The maximum strain of longitudinal steel bars according to the vehicle velocity when the vehicle passes through the bus pad at a constant velocity is analyzed, and the results are shown in Figure 27. The vehicle velocities were set to 20 km/h, 30 km/h, and 40 km/h, and three experiments were performed for each, and the measured values showed slight differences depending on the experiment. Within the range of vehicle velocities considered in the experiment, when compared to the maximum strain of the steel bar at the crack location, the strain is reduced by approximately 10–16% in the steel bar located 0.05 m away from the crack, approximately 43–45% in the steel bar located 0.10 m away from the crack, and approximately 55–60% in the steel bar located 0.15 m away from the crack. This means that the bond between concrete and steel bars in the cracked area is excellent, as already explained in the analysis of the behavior of steel bars under environmental loads.

**Figure 28:**
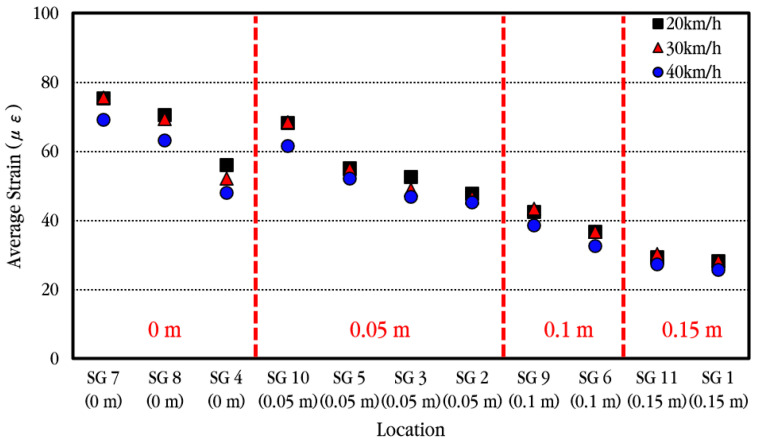
Average maximum dynamic steel bar strains depending on vehicle velocities.

In order to more easily compare a strain change in longitudinal steel bars according to vehicle velocity, the average values of the experimental results performed at the same velocity were calculated and shown in Figure 28. As the vehicle velocity increases, the strain on the steel bar appears to decrease very slightly, but overall, it is analyzed that the strain on the steel bar is not much affected by changes in vehicle velocity.

**Figure 29:**
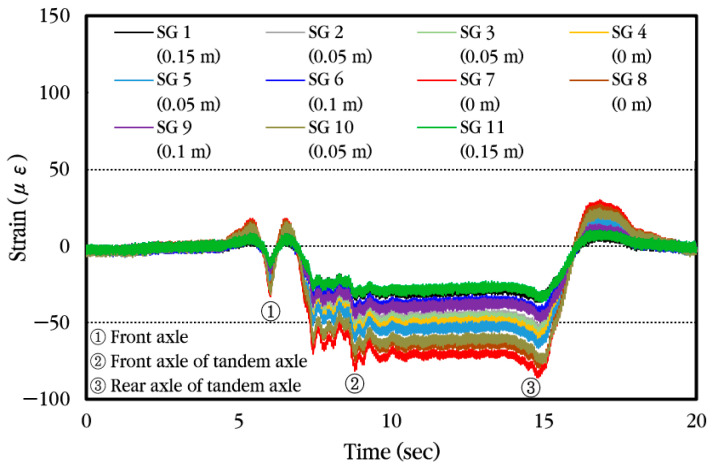
Dynamic steel bar strain history when the vehicle is moving and stopping.

Next, the dynamic strain histories occurring in different locations of the steel bars are analyzed and shown in Figure 29 when the vehicle gradually decelerates upon entering the bus stop and stops so that the front axle of the tandem axle is positioned at the induced crack location where the steel strain gauges are installed and then starts moving again. It can be seen that when the front axle of the vehicle approaches the strain gauge location, tensile strain occurs first at the top of the steel bar, and when the front axle is positioned at the strain gauge location, compressive strain occurs. Afterwards, when the tandem axle approaches the strain gauge location, tensile strain occurs again, and when the front axle of the tandem axle is positioned at the strain gauge location, compressive strain occurs. When the vehicle moves again and the rear axle of the tandem axle is positioned at the strain gauge location, compressive strain becomes maximum. Finally, when the tandem axle passes through the strain gauge, tensile strain occurs again at the top of the steel bar, and then the strain becomes zero.

**Figure 30:**
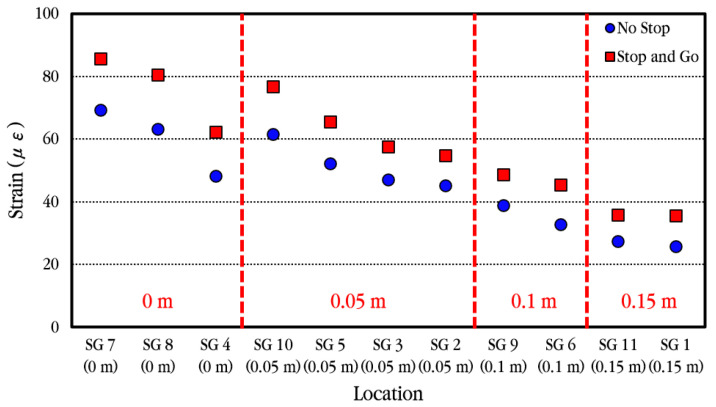
Average maximum dynamic steel bar strains depending on the vehicle movement case.

Figure 30 compares the maximum strains of the longitudinal steel bars according to the distance from the induced crack when the vehicle stops at the crack location temporarily and when the vehicle moves without stopping. In general, the strain change patterns of the steel bars according to the distance from the crack are similar, but it can be seen that the maximum strains of the steel bars increase somewhat when the vehicle stops at the crack location temporarily and then moves. Therefore, it is analyzed that when a vehicle passes through the CRC bus pad, the strain of the steel bar is affected to some extent by the acceleration and deceleration of the vehicle.

## 5. Discussion

### 5.1. Performance of CRC Bus Pad Under Environmental Loads

The early-age performance is evaluated by analyzing various behaviors of the CRC bus pad under environmental loads. As can be seen from the design of CRCP, the most important factors for ensuring the long-term performance of CRCP are the stress of steel bars, the size of crack width, and the crack pattern. Since the long-term performance of CRCP generally depends on its early-age performance [47,52], the early-age performance of the CRC bus pad must be excellent. In this study, using steel strain gauges, the strains of steel bars in the cracked area were measured and converted into stresses. As a result, it was confirmed that the maximum stress was approximately 50 MPa, which was significantly smaller than the yield strength of the steel bar, which is 400 MPa. In addition, it was confirmed that the strain on the steel bar was significantly reduced even at a distance of 0.15 m from the crack, which means that the bonding performance between concrete and steel bars was excellent. Therefore, under environmental loads, the stress level acting on the longitudinal steel bars and the bonding performance between concrete and steel bars were evaluated to be appropriate for ensuring excellent performance of the CRC bus pad.

**Figure 31:**
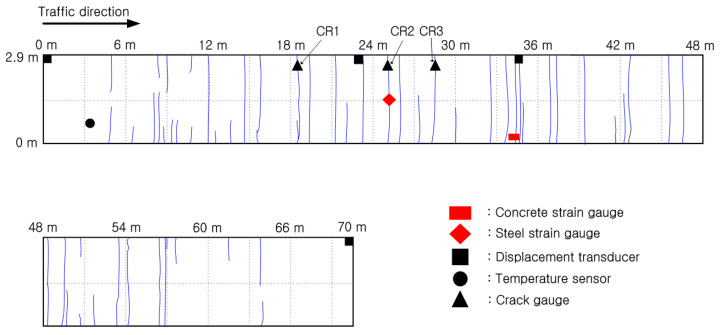
Early-age crack occurrence in CRC bus pad.

As a result of analyzing the crack width change using crack gauges, it was confirmed that the crack widths were less than about 0.3 mm, which was evaluated as appropriate because it was less than the crack width design standard of 1 mm [48,55]. Additionally, the crack occurrence pattern was analyzed as shown in Figure 31. Since it was the early stage of construction, cracks often occurred only partially, and the cracks occurred in almost straight lines, so irregularly shaped cracks such as inclined cracks, D-shaped cracks, and zigzag cracks that reduce the performance of the CRC bus pads did not occur. Therefore, the CRC bus pad was evaluated to have excellent early-age performance with respect to cracks. In addition, both the horizontal and vertical behaviors of the CRC bus pad were very small, and it was evaluated that these behaviors would not reduce the performance of the CRC bus pad.

### 5.2. Performance of CRC Bus Pad Under Vehicle Loads

The dynamic responses of the CRC bus pad due to moving vehicle loads were measured using concrete and steel strain gauges, and the collected data were analyzed to evaluate its performance. The dynamic strain of the concrete slab was analyzed considering various moving conditions of the vehicle, and the measured maximum strain was 254.6 με. If the elastic modulus of concrete of 27.5 GPa is simply multiplied by the strain to predict the tensile stress of the slab, it is approximately 7 MPa. This can be judged as a very large stress being applied, as it is higher than the flexural tensile strength of the concrete slab, which is about 5 MPa. However, in this experiment, a crack occurred at the location of the concrete strain gauge, and the strain gauge measured the opening and closing displacements of the crack, not the strain of the slab. Therefore, a finite element analysis model was created [56], and analysis was performed to convert the measured values of the strain gauge into slab strain in the absence of the crack.

**Figure 32:**
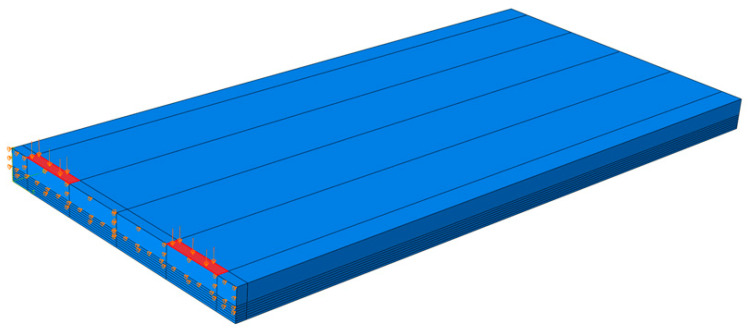
Overview of structural analysis model of cracked CRC bus pad.

As shown in Figure 32, the concrete slab of the finite element analysis model was composed of solid elements and was modeled with dimensions of 12 m in the longitudinal direction, 2.9 m in the transverse direction, and 0.3 m in thickness. To avoid modeling a long CRC bus pad, a sensitivity analysis on slab length was performed to find the minimum slab length that does not affect the stress at the loading location. Based on the results of the sensitivity analysis, the longitudinal length of the slab was selected as 12 m. Since a symmetric model was used for the analysis, the actual longitudinal length of the model was 6 m. The elastic modulus and Poisson’s ratio of concrete were assumed to be 27.5 GPa and 0.15, respectively. The longitudinal steel bars inside the slab were composed of wire elements and were arranged in the same manner as the reinforcement design of the CRC bus pad. The elastic modulus and Poisson’s ratio of the steel bars were assumed to be 200 GPa and 0.3, respectively. The support layer of the slab was simulated as an elastic foundation, and the stiffness was assumed to be 30 MPa/m. The vehicle load was simulated based on the vehicle used in the experiment, and the red rectangles in the figure represent the load contact areas, which are 0.3 m in the transverse direction and 0.2 m in the longitudinal direction, respectively. A distributed load of 1.000417 N/mm^2^ per load contact area was applied based on the load magnitude of the front axle of the tandem axle, which has the largest load ratio. The existence of a crack was simulated at the bottom of the slab, and the crack occurrence length was varied to 0.02 m, 0.04 m, 0.06 m, 0.08 m, 0.10 m, 0.12 m, and 0.14 m from the bottom of the slab to perform the analysis. As shown in Figure 32, the longitudinal displacement at the plane of symmetry, which is the center of the slab, is constrained as a boundary condition. If a particular region of the plane of symmetry allows longitudinal displacement, that region is a crack region.

**Figure 33:**
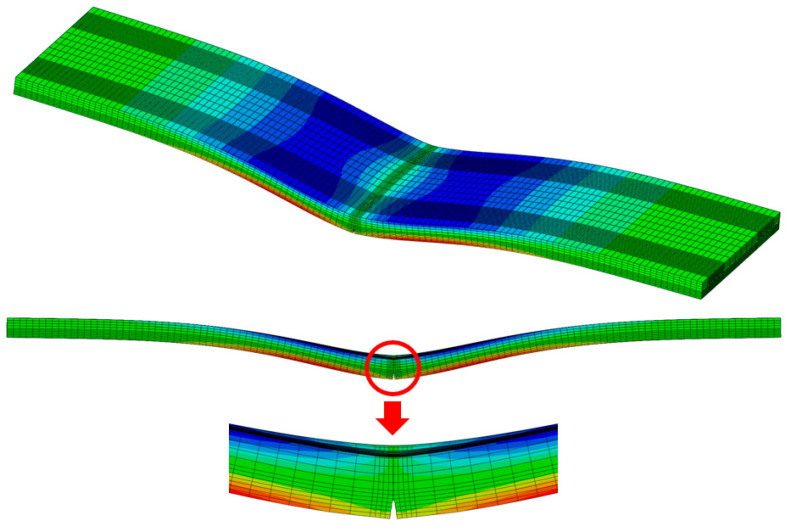
Deflected shape and stress distribution of cracked CRC bus pad.

As shown in the example in Figure 33, finite element analysis was performed according to the length of the crack that occurred in the slab, and the horizontal displacement was analyzed at a location 0.02 m from the bottom of the slab, where the concrete strain gauge was installed. Using the analyzed displacement, the strain of the slab without cracks was predicted by applying Equations (1)–(3). As can be seen in the equation, the displacement obtained from the finite element analysis was first converted into strain by dividing it by the gauge length of 0.1 m of the concrete strain gauge, and the strain ratio was calculated based on the case where no crack occurred. The maximum strain of the concrete slab, 254.6 με, obtained from the experiment was divided by the strain ratio to calculate the slab strain at the location of the concrete strain gauge according to the crack occurrence length, and it was multiplied by the elastic modulus of concrete to predict the stress. Table 2 summarizes the relevant information for predicting these strains and stresses.

(1) $${Converted\;strain}_{Crack\;length=x}=\frac{Measured\;strain}{{Strain\;ratio}_{Crack\;length=x}},$$

where

(2) $${Strain\;ratio}_{Crack\;length=x}=\frac{{Strain}_{Crack\;length=x}}{{Strain}_{No\;Crack}},$$

(3) $${Strain}_{Crack\;length=x}=\frac{{Analysis\;displacement}_{Crack\;length=x}}{Gauge\;length}.$$

**Figure 34:**
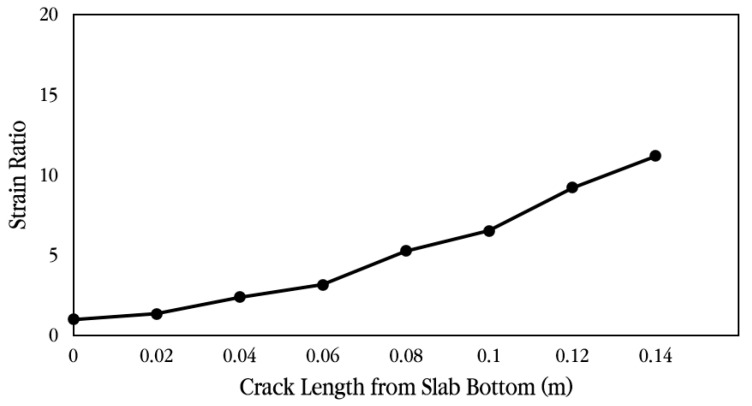
Strain ratio according to crack length from slab bottom.

**Figure 35:**
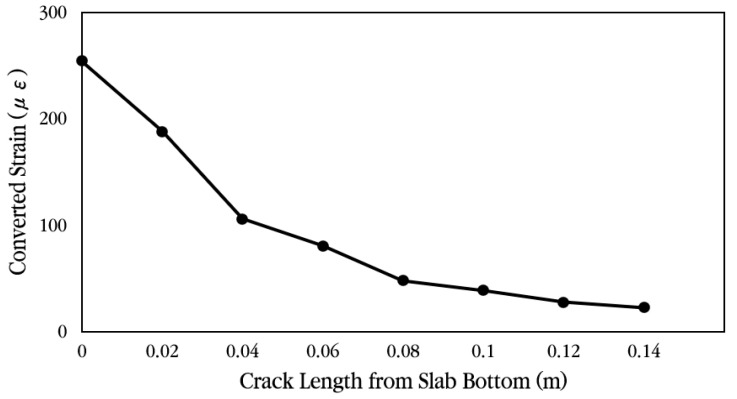
Converted strain according to crack length from slab bottom.

Figure 34 and Figure 35 show the strain ratio and the converted strain according to crack occurrence length from the slab bottom, respectively. When a crack exists at the bottom of the slab, if the measured strain is converted to the strain when there is no crack in the slab, it can be seen that the strain is greatly reduced. For example, if there is no crack in the slab, the measured strain is the actual strain of the slab, but if there is a 0.04 m long crack at the bottom of the slab, the measured strain must be divided by the strain ratio 2.40, as shown in Table 2, to obtain the strain of the slab without a crack. Therefore, in this experiment, since a crack exists at the strain gauge location, even if it is assumed that the crack is only 0.04 m long, the tensile stress of the slab is approximately 2.92 MPa, as shown in Table 2, which is significantly smaller than the flexural tensile strength of the concrete slab. In addition, since the stress of the slab becomes very small at about 0.76 MPa when the length of the crack is 0.12 m, it was analyzed that the stress of the slab due to vehicle loads is in a range that does not affect the performance of the CRC bus pad. In particular, if the stress of the slab is less than about 50% of the flexural tensile strength, it is evaluated that fatigue failure does not occur and long-term performance is ensured [57,58].

When a moving vehicle load is applied to the CRC bus pad, the dynamic strain of the longitudinal steel bar is largest at the crack location, and it is confirmed that the steel bar strain decreases significantly as it moves away from the crack due to the excellent bond between concrete and steel bars [59]. In addition, it is confirmed that the strain of the steel bar is hardly affected by the vehicle velocity, as the difference in strain according to velocity is minimal in the vehicle velocity range used in this experiment. The maximum strain of the steel bar obtained in this experiment, 86 με, is converted to steel bar stress, which is approximately 17 MPa, and it is analyzed that this is significantly smaller than the yield strength of the steel bar, 400 MPa. Therefore, it is confirmed that the stress applied to the steel bars of the CRC bus pad by the moving vehicle is within a range that does not cause a decrease in the performance of the bus pad.

## 6. Summary and Conclusions

This study was conducted to analyze the behavior and performance of pavement in a section where cast-in-place CRCP was applied to the bus stop pavement of a central bus-only lane in an urban area. Using various measurement sensors, the concrete slab strain, steel bar strains, horizontal and vertical displacements, and crack behavior of the CRC bus pad due to environmental loads were measured, and the dynamic responses of the concrete slab and steel bars due to moving vehicle loads were also measured. By analyzing these measurement data, the behavior of the CRC bus pad was identified, and its performance was evaluated along with finite element analysis. The important findings from this study are provided as follows:

- Under environmental loads, the daily strain change in steel bars occurs largest at the crack location, and a strain change in steel bars decreases significantly as it moves away from the crack, which means that the bond between concrete and steel bars is excellent. If the maximum strain of steel bars is converted to the stress, it is significantly smaller than the yield strength of the steel bar. Therefore, the range of stress acting on the steel bars under environmental loads is analyzed to be appropriate for ensuring excellent performance of the CRC bus pad.
- The crack widths are analyzed to be much less than the crack width design standard. In addition, the result of analyzing the crack occurrence patterns shows that the cracks occur in almost straight lines, and irregularly shaped cracks do not occur. Therefore, the CRC bus pad is evaluated to have excellent early-age performance with respect to cracks. Since a crack occurred at the location of the concrete strain gauge in this experiment, a method to convert the measured values of the strain gauge into strains of the slab without a crack is proposed using a finite element analysis. The analysis results show that the stress of the slab due to vehicle loads is very small and is within a range that does not affect the performance of the CRC bus pad.
- The vertical displacements of the CRC bus pad under environmental loads are largest at the entry and exit sections and decrease toward the center. However, since the magnitude of the vertical displacement is very small despite the considerable length of the CRC bus pad, it is evaluated that the curling phenomenon of the CRC bus pad will not have a negative effect on the pavement performance. The longitudinal displacements of the CRC bus pad under environmental loads are also very small even at the entry and exit sections due to frictional resistance with the underlying layer. Therefore, the longitudinal displacement of the CRC bus pad will not affect the pavement performance.
- Within the vehicle velocities used in this experiment, up to 40 km/h, the strain of the slab is almost independent of the vehicle velocity and the vehicle acceleration and deceleration. The strain of steel bars as the vehicle moves with a constant velocity is almost independent of the vehicle velocity. However, the strain on steel bars increases somewhat when the vehicle stops at the crack location temporarily and then moves. Therefore, it is concluded that when a vehicle passes, the strain of steel bars is affected to some extent by the acceleration and deceleration of the vehicle.
- The strain of steel bars of the CRC bus pad subjected to moving vehicle loads is largest at the crack location and decreases significantly as it moves away from the crack. If the maximum strain is converted to steel bar stress, it is significantly smaller than the yield strength of the steel bar. Therefore, it is confirmed that the stress applied to the steel bar of the CRC bus pad by the moving vehicle is within a range that does not cause a decrease in the performance of the bus pad.

As analyzed in this study, the durability of pavement can be improved by applying cement concrete pavement to sections of urban areas where asphalt concrete pavement damage is severe. In addition, it is expected that the pavement performance can be further improved by applying CRCP to minimize frequent maintenance work due to joint damage and cracks that commonly occur in JCP.

## Figures and Tables

**Table 1:** Dynamic moving load test method.

Measurement Item	Testing Method	Vehicle Velocity (km/h)
Concrete strain	Vehicle passing gauge location with constant velocity	20, 30, 40
	Vehicle moving in and stopping at gauge location temporarily and moving out	40
	Vehicle passing 0.3 m from gauge location with constant velocity	20, 40
Steel strain	Vehicle passing gauge location with constant velocity	20, 30, 40
	Vehicle moving in and stopping at gauge location temporarily and moving out	40

**Table 2:** Slab stress and strain prediction according to crack length.

Crack Length (m)	Analysis Displacement (mm)	Analysis Strain (με)	Strain Ratio	Converted Strain (με)	Converted Stress (MPa)
0	6.63 × 10^−3^	66.31	1.00	254.60	7.00
0.02	8.96 × 10^−3^	89.59	1.35	188.42	5.18
0.04	1.59 × 10^−2^	159.10	2.40	106.11	2.92
0.06	2.09 × 10^−2^	209.43	3.16	80.61	2.22
0.08	3.49 × 10^−2^	349.17	5.27	48.35	1.33
0.10	4.33 × 10^−2^	432.90	6.53	39.00	1.07
0.12	6.11 × 10^−2^	610.81	9.21	27.64	0.76
0.14	7.43 × 10^−2^	743.22	11.21	22.71	0.62

## Data Availability

The original contributions presented in this study are included in the article. Further inquiries can be directed to the corresponding author.
